# Starting the conversation: A new path in suicide prevention

**DOI:** 10.1017/gmh.2025.10064

**Published:** 2025-10-01

**Authors:** Erin Gallagher

**Affiliations:** Founder, Jay Walkers, Chesterfield, VA, USA

**Keywords:** suicide prevention, suicide stigma, suicide literacy, storytelling, preemptive conversations

## Abstract

Suicide remains a global public health crisis, claiming over 800,000 lives each year and leaving millions more to struggle with attempts, ideation, or the ripple effect of loss. Traditional prevention strategies often focus on crisis intervention and identifying “warning signs,” but these approaches overlook the many who suffer in silence. Drawing on personal experience of suicide loss and a decade-long journey toward suicide literacy, the author argues for a reframing of suicide prevention. She challenges stigma-driven assumptions, underscores the power of honest storytelling, and introduces the concept of “preemptive, protective conversations” as a vital upstream prevention tool. By empowering ordinary people to become suicide prevention advocates equipped with knowledge, compassion, and a willingness to talk openly, we can build stronger connections, dismantle stigma, and create a broader societal safety net. Suicide is preventable, and each of us has a role to play in saving lives.

## The problem

Suicide is a significant global public health concern. We lose approximately 800,000 people worldwide to suicide each year (WHO, 2019). In the United States, despite a quarter-century of dedicated research and prevention initiatives, annual suicide numbers have increased 30% from 2000 to 2022, with notable exceptions from 2018 to 2021 (Garnett and Curtin, 2024).

The scope of the US challenge is not limited only to losses. For every person who dies by suicide in the United States, there are 32 others who have attempted, 76 who have made plans, and 264 who experience ideation. That amounts to 18.65 M people whose lives are altered by suicide each year (SAMHSA, 2023).

The annual cost of suicide in the United States is estimated to be $510B (Peterson et al., 2024).

## The human impact

For most readers of this article, the suicide statistics cited above are not surprising. As advocates in suicide prevention, we repeat these numbers so frequently that we may disassociate them from the human loss connected to them, the actual people who once were here but now are not, who leave behind an unfillable hole and a wake of sadness.

It is often asserted that when a person dies by suicide, six others are impacted. A 2014 study (Cerel et al., 2019) suggests the real ripple effect of suicide is more than 20 times greater. For each person who dies by suicide, as many as 135 others are impacted. This means that, annually, around the globe, 105 million people must contend with the tragic loss of a loved one, friend, neighbor, or colleague and the shock, grief, and heartbreak that follows.

For too many of us, suicide is a deeply personal subject.

## Our story

I lost my son, Jay, to suicide in 2016. Jay was a bright, thoughtful, and funny high school senior who was engaged in academics and after-school activities. He was a leader among his peers and was making plans for his future. Then, he died by suicide. Jay’s death was a traumatic shock to our family and our community.

Following his death, in my profound grief, I embarked on a journey to understand how suicide could happen to Jay, or to anyone. I took suicide awareness training, attended information sessions, pored over research articles, and read many books to understand the topic better. I took a job in the field of mental health awareness where I connected with suicide attempt survivors. Throughout it all, I was motivated by a deep desire to comprehend suicide, how it happens, and what can be done to prevent it.

In my journey toward suicide literacy, I pushed myself to imagine the depths of despair that Jay must have been experiencing right before his death. That exercise helped me draw some important conclusions about Jay’s experience. First, Jay did not know that suicidal ideation is a fairly common experience (SAMHSA, 2023). He did not know that suicidal ideation is not always a sign of mental illness (Leenaars, 2010). He did not know that many people go on to live well and thrive after experiencing suicidal thoughts or even after attempting to take their own lives (Gibb et al., 2005).

Unfortunately, before Jay died, I did not know this information either. But now I do. The knowledge I have acquired has driven me to become a suicide prevention advocate, eager to share what I have learned about suicide with as wide an audience as possible, to inspire change in how we protect ourselves and our loved ones from suicide.

## Storytelling saves lives

My first step into suicide prevention advocacy was in storytelling. Most importantly, I told the true story of Jay’s death from the very beginning. When family and friends flinched (and suggested they would not mention the manner of his death to anyone), I insisted that they, too, speak openly. I knew immediately that covering up Jay’s suicide would perpetuate a dangerous silence that put others at risk. Later, I shared about my grief journey, my deep dive into suicide, and Jay’s story in blog posts, speaking engagements, and conference presentations.

I know that being open and honest about our experience has made a difference in others’ lives. On the day Jay died, family friends sat their children down and implored them, “If you ever, ever feel this way, please talk to us. You must tell us right away so we can get help for you.” Without a second’s hesitation, their teenage son replied, *“*I feel like that every day*.”* His parents were absolutely stunned, but immediately found him the help he needed. Their son is now a thriving young man in his late 20s.

Frequently, my storytelling journey has overwhelmed and exhausted me. But, I tell Jay’s story and mine because I know that storytelling saves lives. I persist even when it is hard because I desperately want to prevent others from experiencing both the darkness that overwhelmed Jay and the everlasting shadow of tragic loss that haunts me and others like me who have endured suicide loss.

## What I have learned

Our friends’ story about their son made me wonder, *if others want to initiate conversations about suicide, how should they begin?* I turned to the Internet in search of helpful resources but was disappointed to find very little guidance.

Even today, years after my first search, the results are disappointing. If you google “how to talk about suicide,” you will primarily find suggestions on how to start a conversation with someone you suspect is at risk of suicide. The typical advice suggests if you notice suicide warning signs in a friend or loved one, you should start a conversation with them. The problem with this “if you see something, say something” approach, as I have learned, is that the process for identifying those “at risk” is inexact.

First, suicidal people do not always show signs. It is widely reported that four in five suicidal people display typical warning signs (NIMH, 2025). A strategy to reach out to them excludes the other 20% (Rodway et al., 2020; Zuromski et al., 2024).

Second, many suicide attempt survivors, when interviewed post-attempt, report that there was very little time – as little as five minutes to two hours – between their first thought of suicide and their attempt (Simon et al., 2001; Deisenhammer et al., 2009), leaving practically no opportunity for a loved one to notice that they were in trouble and initiate a conversation.

Third, many suicidal individuals, even those displaying warning signs, are motivated to keep their plans a secret (Blanchard and Farber, 2015) because of suicide myths and stigma. Even a point blank question about their desire to die could result in a denial.

**Figure 1.**
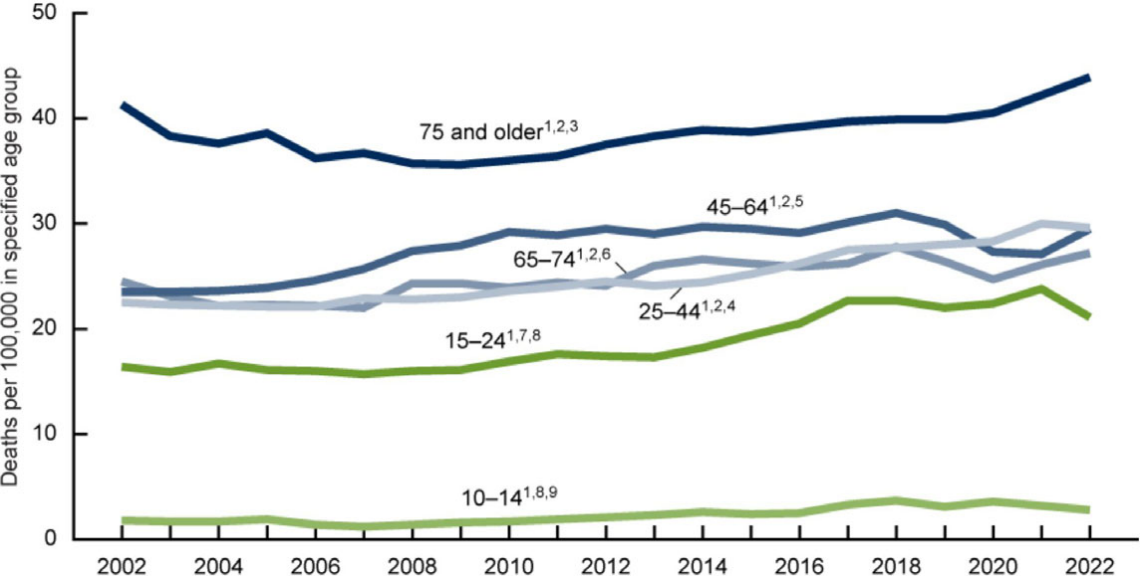
Suicide Rates for males in the U.S. 2002 – 2022.

Next, our ideas of suicide are steeped in misconceptions and a dangerous lack of information. For example, many people assume that suicide is primarily a teen concern (Sharma, 2024). However, in the United States, teens make up only 15% of suicide deaths each year. Who are the other 85%? This 2024 chart from the US Center for Disease Control (Figure 1) helps paint the picture: the group with the highest risk of suicide is consistently men over the age of 75 (Garnett and Curtin, 2024).

There is also an incorrect assumption that suicide is a risk only for those diagnosed with mental illness. However, many people without a diagnosed mental health condition die by suicide (Fowler et al., 2022; D’Arrigo, 2024).

Suicide myths and misconceptions distract us from the reality that suicide can happen to anyone at any stage in life. They may lead us to miss signs in some because we have concluded erroneously that they are not at risk. Suicide risk actually increases over a person’s lifetime and can become more acute with the convergence of various social, emotional, economic, or health stressors (Stack, 2021).

Finally, this strategy seems to overlook the millions of people who live with suicidal thoughts, but who may not yet (or ever) be at a breaking point (Samples et al., 2024). Is their suffering not worth our intervention? One study seemed to suggest that (Qiu et al., 2017)!

While we refine our understanding of risk, we continue to face a steady drumbeat of unrelenting suicide loss in our communities. Globally, we lose one person every 40s (WHO, 2019). More people die by suicide worldwide than by violent crime (Schlein, 2014). Isn’t there something more we could do to save lives?

## Suicide stigma

The information on when to start conversations about suicide seems to be heavily influenced by stigma. Stigma refers to the mark that society places on an individual because of perceived differences, either seen (such as body shape or use of a prosthetic or wheelchair) or unseen (such as mental illness). Public stigma around mental health leads to the marginalization of an individual in one or more ways including labeling, stereotyping, and discrimination (Link and Phelan, 2001).

Individuals are sensitive to public stigma and often internalize it (Corrigan and Watson, 2002). A person with internalized stigma may avoid revealing their experiences or self-isolate to avoid being labeled, shamed, or judged.

Structural stigma arises when public and personalized stigma are codified into laws, policies, and practices (Link and Phelan, 2001).

Suicide evokes both public and internalized stigma. “Suicide is a crime (Hasnain, 2022)” and “suicide is a sin (Khosa-Nkatini and Buqa, 2021)” are two of the most persistent stigmatizing notions. Others include opinions that someone who takes their own life is weak or selfish. These public attitudes about suicide and those who succumb to it influence the behavior of people who experience suicidal thoughts. Suicidal people with internalized stigma may hesitate to reveal their mounting crisis to others and may avoid seeking treatment out of fear of being labeled or shamed.

It is clear that public and personal stigma stand in the way of people asking for – and offering – lifesaving help. The current guidance suggesting we start conversations only in the most dire situations is systemic stigma that frustratingly reinforces commonly held beliefs that we should not talk openly about suicide, unless, of course, it is absolutely necessary.

## Reframing suicide

Jay’s birth in 1997 made me a first-time mother. I reveled in this role and our connection. Every twinkle of his eye, every giggle – or later, every deep-throated chuckle – made my heart swell with love and pride. This is my son! I get to be his mom!

So when he died by suicide, I was overwhelmed by grief and the sting of suicide stigma for a very long time. In the first few months, I deeply grieved his absence, not only in that he was no longer physically present but also in that our treasured, soul-level bond now felt permanently severed. I spent time in therapy to try to heal the wound of Jay’s self-inflicted death. I could not help but see it as a deliberate act of destruction, not just of him but of us. I did not want to judge him, but I could not avoid it.

My therapist suggested I tell myself a new story, one that reframed Jay’s suicide. I gained initial traction on this front with the realization that the human brain is an organ, like the heart or the kidneys. I could acknowledge that hearts and kidneys fail sometimes, and that can lead to loss of life. So, I had to acknowledge that brains fail too, and that failure can also lead to death. That explanation, albeit over-simplified, resonated with me. I wrote it down and re-read it frequently until it permeated my conscience and delivered the first chink in the suicide stigma that was upsetting me.

This tiny shift motivated me to find other ways to reframe Jay’s suicide. One morning, I attended an online suicide prevention webinar presented by John Sommers-Flanagan, PhD, of the University of Montana. The very first slide moved me to tears. It said, “De-pathologize and view suicide disclosures as a natural communication of pain and an unmatched opportunity to offer compassionate help.”

I had never heard anyone talk about suicide this way. To eject shame from suicidality and instead frame it as *a natural communication of pain*, to honor someone’s confession of suicidal thoughts as an *opportunity for compassionate help*, to *de-pathologize* suicide? What a gift this perspective was to me.

Later, I read *Rethinking Suicide: Why Prevention Fails and How We Can Do Better*, by Craig J. Bryan, PsyD, ABPP, and finally became convinced that much of what I thought I knew about suicide was wrong. *Rethinking Suicide* challenges the status quo of suicide prevention and offers an innovative plan for prevention in the future. “To do suicide prevention well,” Dr. Bryan suggests, “we have to do more than just stop people from dying -- we also have to make life worth living.” (Bryan 2022)

In my advocacy work, I have had the privilege of hearing others’ stories of suicide (Breel, 2013; Fiorello, 2015; Wright, 2019) These storytellers articulate real pain, suffering, and inner conflict, but they also express themselves with sophistication, humor, and invaluable insight. And they offer me a much more complex picture of suicide than the one my stigmatized mind had crafted.

I am grateful for having been exposed to these expert and storyteller perspectives. My own stigma has drastically diminished because of them.

## New attitudes

Public perceptions of suicide seem to be improving, too.

In a recent Harris Poll (2024) on beliefs and attitudes about mental health and suicide in the United States, for example, 91% of respondents said they believe that suicide can be prevented at least some of the time. Ninety-four percent said they would take action if someone close to them was thinking of suicide. Seventy-two percent of respondents would like to see more professional trainings available, and 56% would like opportunities for the public to be educated about suicide prevention.

These results are extremely promising as they suggest a growing desire for suicide literacy and a willingness on the part of ordinary people to personally engage in suicide prevention efforts. This presents a significant opportunity to introduce an additional layer of protection to the standard slate of suicide prevention efforts.

## Prevention advocates

Recent publications from the World Health Organization (2021) and the US Department of Health and Human Services (2024) suggest that suicide prevention is the work of communities, not just clinicians. This guidance represents a call to action for ordinary people to participate actively in suicide prevention efforts. Why do we not encourage individuals to take on the identity of suicide prevention advocates and start preemptive, protective conversations in their circles?

Those conversations could be as simple as the one my friends had with their children the day Jay died. They had no suspicion that either child was struggling, yet they still sat them down to discuss suicide, just in case. The strategy worked. Their message cleared the way for their son to reveal his feelings.

The benefits of initiating conversations as a prevention advocate are numerous. Most importantly, preemptive conversations represent an important upstream shift away from crisis intervention toward the prevention of not only suicide but also unnecessary suffering.

These conversations will strengthen suicide literacy in families and social circles by opening the door to acknowledge in a judgment-free manner that despair, hopelessness, and suicidal thoughts can happen to anyone over the course of a lifetime. They will reduce stigma by creating opportunities to dismantle suicide myths and replace them with data-driven and evidence-based facts. And they allow families and friends to devise plans for how to safely respond should any such experiences arise. Most importantly, preemptive conversations strengthen relationships, and stronger relationships, in turn, offer greater protection against suicide (Van Orden et al., 2010; Feeney and Collins, 2015; AIFS, 2024; Stoms et al., 2025).

Enlisting ordinary people as suicide prevention advocates to start preemptive, protective conversations about suicide is an evidence-informed strategy (Ibrahim, 2023; Jahan et al., 2023; Žilinskas and Lesinskienė, 2023) and represents an accessible upstream prevention effort that can be enacted and grown at the grassroots level. This form of prevention requires little investment up front, yet it holds the potential for great impact. It offers an additional layer to existing prevention efforts, one that starts small but whose ripple effects when adopted at scale can transform communities and society.

## Jay walkers for suicide prevention

This year, I launched a non-profit in Jay’s memory called Jay Walkers. At Jay Walkers, we envision a world where suicide is no longer a public or personal health crisis. Our mission is to empower individuals to take steps to prevent suicide through education and action. We engage individuals with a simple but profound call to action: connect to the Jay Walkers community and mission, bolster your suicide literacy, and then contribute to the overall solution as suicide prevention advocates.

The central campaign of Jay Walkers is the annual Jay Walkers Challenge in September. The challenge invites teams of Jay Walkers to work together to complete 800,000 steps over the course of the month. When they do, they will have walked one step in honor of every life lost worldwide annually to suicide. Teams also complete challenge activities to support our mission, including:

Starting Conversations: Participants will start preemptive and protective conversations about suicide with friends and family. Jay Walkers are encouraged to share their own stories and/or let others know it is safe to share theirs. We offer training to help participants build their confidence and skills in this area.

Taking More Steps: After the challenge, participants are encouraged to continue on the path toward full-fledged suicide prevention advocacy in order to benefit themselves, their loved ones, and their communities.

The challenge sets the stage for individuals to contemplate more deeply their relationship to and attitudes about suicide. We then work to spark participants’ interest in becoming full-fledged suicide prevention advocates focused on increasing suicide literacy within their own family and social circles. Down the line, Jay Walkers will seek more evidence-based suicide prevention efforts for our community of suicide prevention advocates to support and promote.

As the Jay Walkers community grows, the modest individual contributions of our community members will add up to significant impact. Jay Walkers will demonstrate that together we can effectively strengthen the societal suicide safety net that leads to a reduction in suicide rates worldwide.

## Conclusion

It has been almost 10 years since I lost Jay. My journey to suicide literacy has taught me so much. Oh, how I wish I knew then what I know now! Maybe things would have been different for Jay.

In recent years, my pain around Jay’s death has transformed into righteous anger and determined focus. I am heartbroken by stories of suffering and suicide loss. It hurts me deeply to imagine that millions of people are living with an unbearable pain that threatens to consume them. And I am frustrated that suicide stigma continues to prevent us from confronting the reality of suicide for the safety of our family and loved ones. There are too many people in the world right now suffering in silence. Some are sliding closer and closer to their breaking point. What if one of them is someone you love? Imagine the gift a preemptive conversation would offer them.

I have seen the power of one story and one conversation. And that has unlocked within me a clear vision of a more hopeful future – one in which ordinary people, motivated by love and empowered by newfound knowledge of what suicide is and what suicide is not, take on the mantle of suicide prevention advocacy and start preemptive, protective conversations. Based on current suicide statistics, I am sure that many of those conversations will immediately uncover previously unspoken distress. If not, they will certainly open the door for more vulnerable discussions that result in stronger personal connections that are enriching and protective and may, one day, serve as a lifeline.

Suicide is preventable. Each of us can do our part. When we do, we will build a society where despair is met with understanding, where silence is replaced by healthy conversation, and where lives like Jay’s are protected by the safety net of community.
