# Supplementary material for: Starting the conversation: A new path in suicide prevention
Source: Glob Ment Health (Camb). 2025 Oct 1;12:e118. doi: 10.1017/gmh.2025.10064 (PMC12641319; doi:10.1017/gmh.2025.10064)
Supplement: Peer review history

# Editor report: Recommendation: Starting the conversation: A new path in suicide prevention — R0/PR2

**DOI:** 10.1017/gmh.2025.10064.pr2

**Affiliations:** Social Work, University of South Florida, United States

See my comments below :)

# Editor report: Decision: Starting the conversation: A new path in suicide prevention — R0/PR3

**DOI:** 10.1017/gmh.2025.10064.pr3

**Affiliations:** Social Work, University of South Florida, United States

No accompanying comment.
